# Supplementary material for: How CD4+ T Cells Transcriptional Profile Is Affected by Culture Conditions: Towards the Design of Optimal In Vitro HIV Reactivation Assays
Source: Biomedicines. 2023 Mar 13;11(3):888. doi: 10.3390/biomedicines11030888 (PMC10045592; doi:10.3390/biomedicines11030888)
Supplement: Supplementary file 1 [file biomedicines-11-00888-s001.zip › Supplementary Figure S1.pdf]

Figure S1.

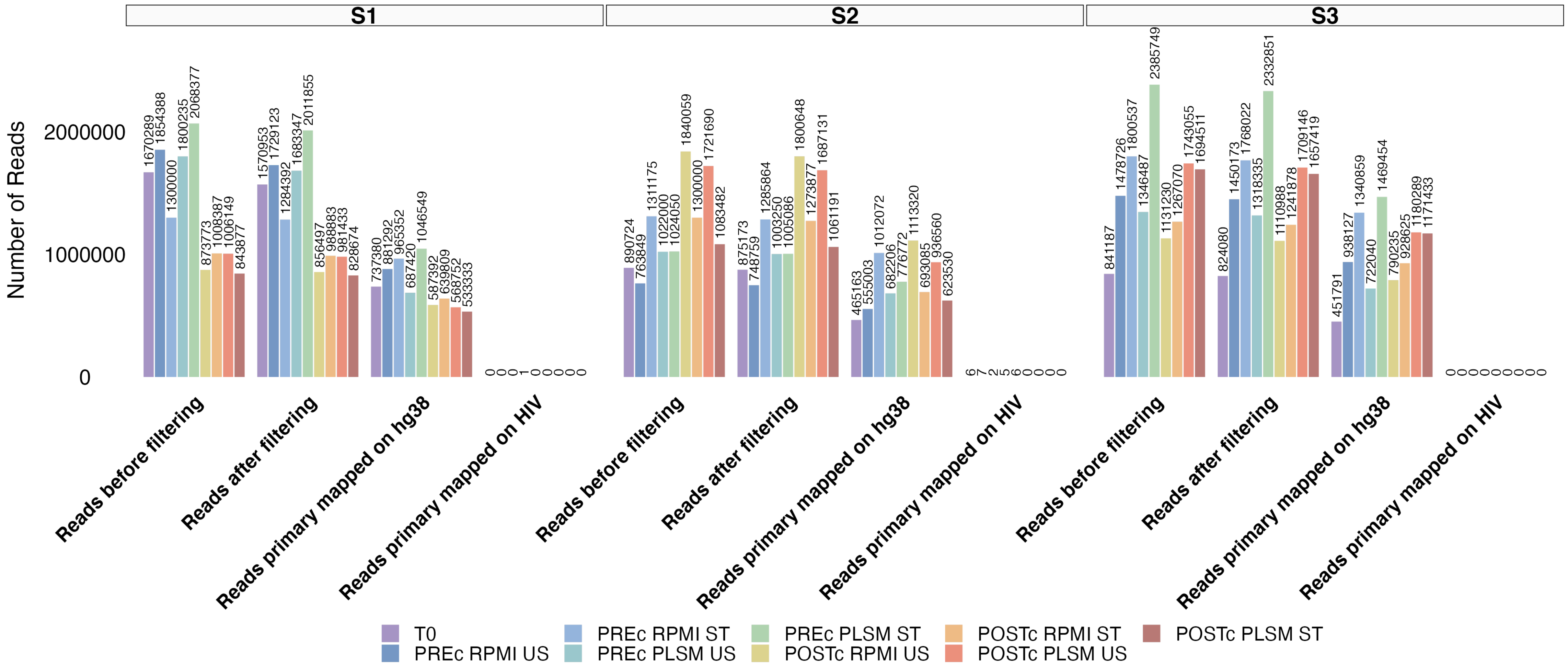

Figure S1. Histogram showing the numbers of total reads sequenced, reads left after filtering (reads with more than 40% of the base with a Phred score of less than 10 were filtered out), 523 reads aligned on the human transcriptome (hg38 Gencode), and reads aligned against the Human 524 immunodeficiency virus type 1 (HXB2) genome sequence.
