# Supplementary material for: How CD4+ T Cells Transcriptional Profile Is Affected by Culture Conditions: Towards the Design of Optimal In Vitro HIV Reactivation Assays
Source: Biomedicines. 2023 Mar 13;11(3):888. doi: 10.3390/biomedicines11030888 (PMC10045592; doi:10.3390/biomedicines11030888)
Supplement: Supplementary file 1 [file biomedicines-11-00888-s001.zip › Supplementary Figure S2.pdf]

Figure S2.

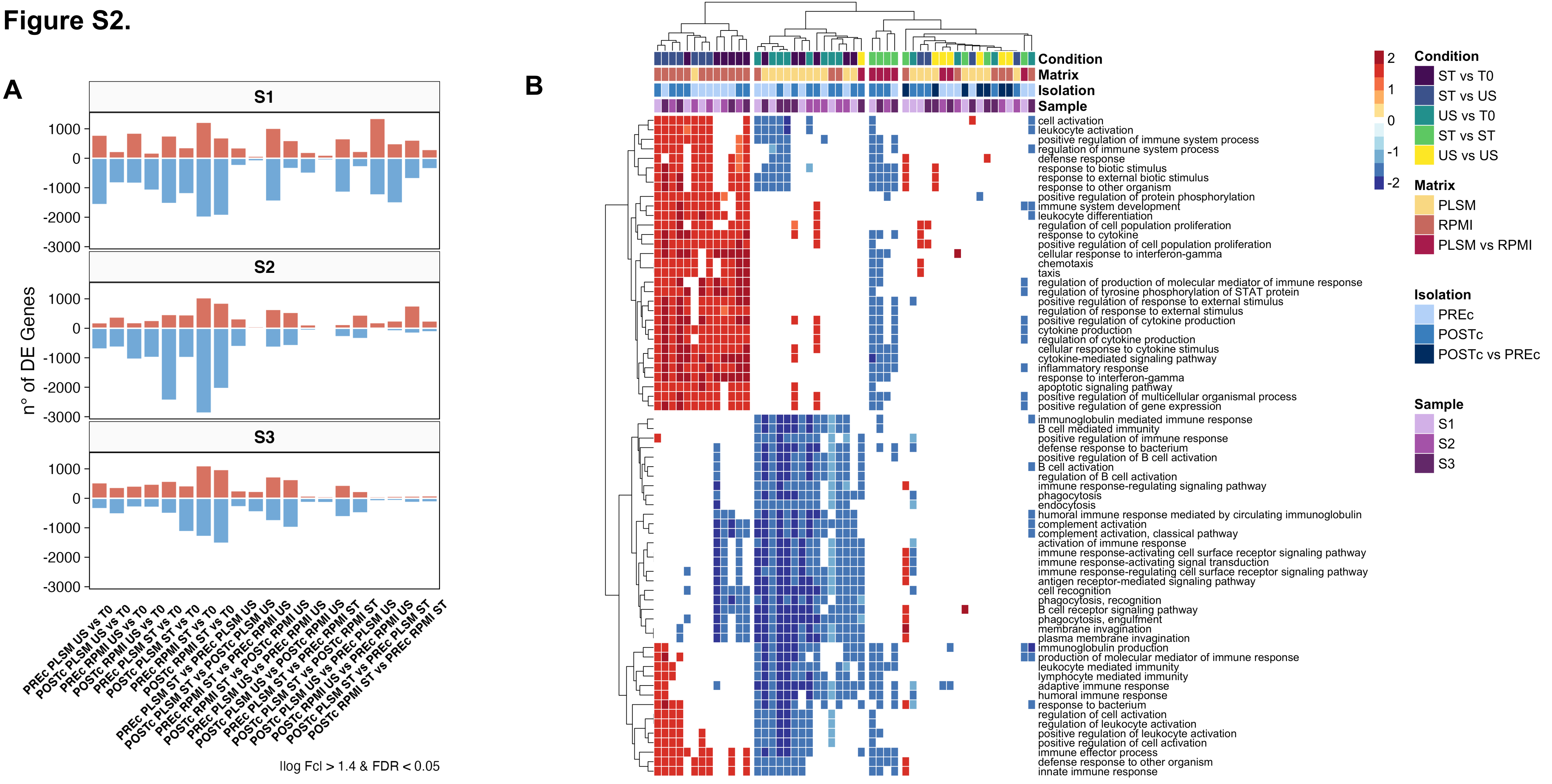

Figure S2. Number of genes up-regulated (red) and down-regulated (blue) between all the 20 comparisons analyzed in the 3 study participants (S1, S2, and S3) (A). Heatmap of NES values from GSEA on biological processes between all the 20 comparisons analyzed. Positive values (red) indicate biological processes enriched in genes up-regulated. Conversely, negative values (blue) show processes enriched in down-regulated genes. White indicates that there are no significant results (A).
