# Supplementary figures and images for: How CD4+ T Cells Transcriptional Profile Is Affected by Culture Conditions: Towards the Design of Optimal In Vitro HIV Reactivation Assays
Source: Biomedicines. 2023 Mar 13;11(3):888. doi: 10.3390/biomedicines11030888 (PMC10045592; doi:10.3390/biomedicines11030888)

Figure S3.

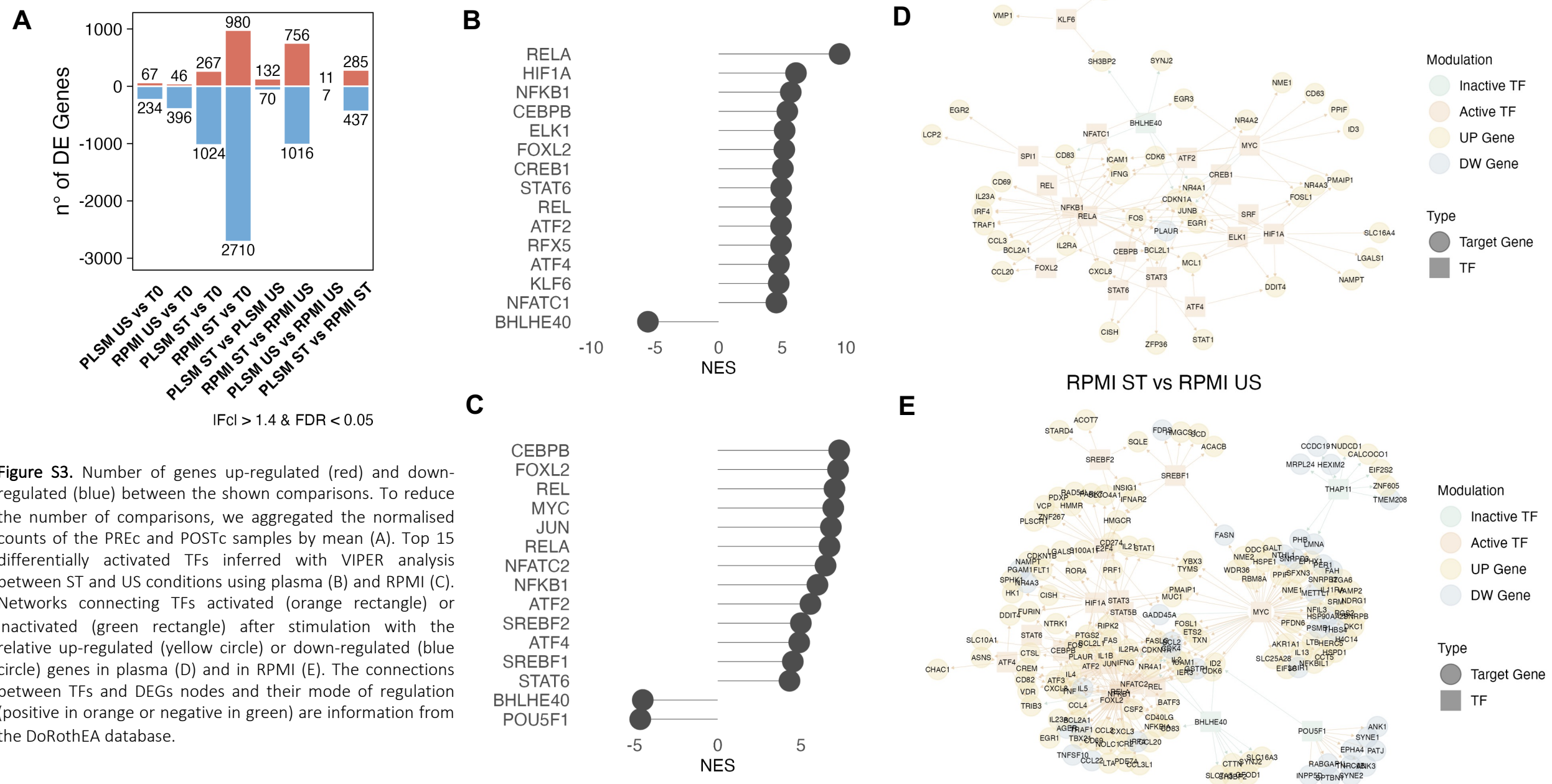

Supplement: Supplementary file 1 [file biomedicines-11-00888-s001.zip › Supplementary Figure S3.pdf]
